# Psychoeducation for depression, anxiety and psychological distress: a meta-analysis

**DOI:** 10.1186/1741-7015-7-79

**Published:** 2009-12-16

**Authors:** Tara Donker, Kathleen M Griffiths, Pim Cuijpers, Helen Christensen

**Affiliations:** 1 Department of Clinical Psychology, VU University, van der Boechorstraat 1, 1081 BT Amsterdam, the Netherlands; 2 Centre for Mental Health Research, The Australian National University, Building 63 Eggleston Road, ACTON ACT 0200, Canberra, Australia

## Abstract

**Background:**

Given the high prevalence and burden associated with depression and anxiety disorders and the existence of treatment barriers, there is a clear need for brief, inexpensive and effective interventions such as passive psychoeducational interventions. There are no published meta-analyses of the effectiveness of passive psychoeducation in reducing symptoms of depression, anxiety or psychological distress.

**Methods:**

Cochrane, PsycInfo and PubMed databases were searched in September 2008. Additional materials were obtained from reference lists. Papers describing passive psychoeducational interventions for depression, anxiety and psychological distress were included if the research design was a randomized controlled trial and incorporated an attention placebo, no intervention or waitlist comparison group.

**Results:**

In total, 9010 abstracts were identified. Of these, five papers which described four research studies targeting passive psychoeducation for depression and psychological distress met the inclusion criteria. The pooled standardized-effect size (four studies, four comparisons) for reduced symptoms of depression and psychological distress at post-intervention was *d *= 0.20 (95% confidence interval: 0.01-0.40; *Z *= 2.04; *P *= 0.04; the number needed to treat: 9). Heterogeneity was not significant among the studies (*I*^2 ^= 32.77, Q:4.46; *P *= 0.22).

**Conclusions:**

Although it is commonly believed that psychoeducation interventions are ineffective, this meta-analysis revealed that brief passive psychoeducational interventions for depression and psychological distress can reduce symptoms. Brief passive psychoeducation interventions are easy to implement, can be applied immediately and are not expensive. They may offer a first-step intervention for those experiencing psychological distress or depression and might serve as an initial intervention in primary care or community models. The findings suggest that the quality of psychoeducation may be important.

## Background

Depression and anxiety are frequently seen in clinical practice [1] and are associated with personal suffering, reduced quality of life [2] and high economic costs [3]. Despite the availability of effective treatments (pharmacological [4] as well as psychological [5]) in reducing symptoms of common mental disorders, only a minority of people receive psychological treatment delivered by a mental health professional [6]. Several reasons have been proposed to account for the low delivery rate, including the length of waiting lists driven by low workforce numbers [7], the high costs associated with treatment [8], perceived social stigma which reduces help seeking [8] or an inability to identify symptoms of depression [9]. Given the high prevalence and burden associated with these disorders and the existence of treatment barriers, there is a clear need for brief, inexpensive and effective interventions.

Psychoeducational interventions are interventions in which education is offered to individuals with psychological disorders or physical illnesses. For the scope of this review, we focus on psychoeducational interventions for psychological disorders. These interventions can vary from the delivery of 'passive' materials such as single leaflets, emails or information websites [10] to active multi-session group-intervention with exercises and therapist-guidance [11]. Examples of passive interventions are interventions that offer psychoeducational information about disorders or feedback to individuals based on test results or screening tests. Psychoeducational interventions are less expensive, more easily administered and potentially more accessible than conventional pharmacological and psychological interventions. In addition, there is some evidence from systematic reviews that psychoeducational interventions are effective (for example, [12,13]) in treating or preventing mental disorders. However, with the exception of a review of the efficacy of psychoeducational interventions (personalized feedback) for problem drinking [14], these previous reviews have focused on active rather than passive psychoeducational interventions for mental disorders.

The aim of this meta-analysis is to integrate the results of studies evaluating the effectiveness of passive psychoeducational interventions in reducing depression, anxiety or psychological distress compared to no intervention, attention-placebo (for example, a thank you letter or telephone calls informing about the well-being of subjects) or waitlist. More specifically, this meta-analysis examines specific features of psychoeducation that may contribute to its effectiveness. These include the setting in which it is delivered and its content.

## Methods

### Definitions

In this meta-analysis, a passive psychoeducational intervention is defined as an intervention which provides information, education materials or feedback/advice. Examples of passive psychoeducation are programmes offered to individuals through leaflets, posters, audio-visual aids, lectures, internet material or software which aims to educate the recipient about the nature and treatment of depressive and/or anxiety disorders or psychological distress. The intervention can be delivered in primary or secondary care settings, or within universities, community centres or other public venues. Psychoeducation can be delivered through the post, email, via face-to-face lectures or through information published on the web.

Although in some cases it is difficult to distinguish between active and passive education (that is, where encouragement is offered but no explicit instructions are given to carry out certain recommendations), in the present review, passive education was defined as education that did not require the recipient to undertake explicit homework or relaxation exercises and which did not deliver active treatment. Thus programmes which taught the principles or required the implementation of elements of active psychotherapies (for example, cognitive behavioural therapy [CBT] or interpersonal therapy [IPT]) were excluded. Studies were also excluded if psychoeducation was offered in combination with another component, such as CBT or any other broader multifactor intervention.

### Data sources and screening procedures

The Cochrane, PsycInfo and PubMed databases were searched on 25 September 2008, with the key search terms 'depress*' OR 'anxi*' OR 'psychological distress' OR 'mood' OR 'affective' OR '*phobia' OR 'OCD' or 'obsessive compulsive' OR 'panic' AND 'psychoeducation' OR 'education' OR 'information' or 'knowledge' OR 'instruction' OR 'teaching' OR 'mental health literacy' OR 'anxiety literacy' OR 'depression literacy'. In addition, the following limits for retrieving references were applied for the PubMed database: 'humans'; 'clinical trial'; 'RCT'; 'CT phase I to IV'; 'controlled clinical trial'; 'evaluation study'; and 'English'. For the PsycInfo database, the references were limited to: 'humans'; 'treatment & prevention'; 'quantitative study or treatment outcome'; 'randomized clinical trial'; and 'English'. Separate searches for systematic reviews and meta-analyses were done for the PsychINFO and Pubmed database using similar key search terms.

Two independent researchers screened the identified titles and abstracts to determine if the inclusion criteria were met. Full text copies of all potentially relevant papers which met criteria, or papers where there was insufficient information in the abstract to determine eligibility, were retrieved. Full text articles were further screened and excluded from further analysis if inclusion criteria were not met. Reference lists of all included systematic review and meta-analysis studies were checked. The data extraction of relevant papers was completed by two independent researchers, with disagreements resolved through discussion or with a third or fourth researcher.

### Inclusion and exclusion criteria

Studies were included if: the psychoeducation targeted depression, anxiety or psychological distress; participants were described as either experiencing mood or anxiety disorders; or if they experienced elevated scores (equal to or above a specified cut-off score, see Table 1) on depression, anxiety or psychological distress scales. To be included, studies were required to have a randomized controlled design, which incorporated a no intervention, attention-placebo or a waitlist control group to which psychoeducation was compared. All included studies were required to report mental health outcomes (depression, anxiety or psychological distress) and were published in peer-reviewed, English language journals. There was no restriction on the age of participants. Studies were excluded if the education component was offered in addition to other components (for example, psychotherapy with elements of psychoeducation or psychoeducation enhanced with treatment as usual) or when the intervention was compared solely to a (potentially) active treatment (for example, medication, treatment as usual or psychotherapy). Studies were also excluded: when the intervention was not passive psychoeducation but involved an active intervention (for example, components of CBT or IPT, relaxation exercises or homework or group discussion); or when psychoeducation was aimed at target groups where there was a concomitant physical health or mental disorder; or where the target of the intervention was a carer or parent of the person with anxiety or depression (for example, medical illness, other mental health disorders, parental programmes, family-caregiver programmes).

**Table 1:** Psychoeducational studies for depression and/or anxiety.

Study	Aims of study	Study design/intervention	Population	Primary outcome measures	Outcomes of interest	Effect size (cohen's *d)*^a^	JQR^b^
Christensen *et al*. (2004) [10]	To evaluate the efficacy of a psycho- education website	RCT; Blue Pages (*n *= 136) versus attention placebo controls (telephone calls; *n *= 157) Format: individual	Community residents (18 to 52 years) with internet access in Canberra, Australia	Center for Epidemiologic Studies (CES-D).	Compared to controls, intervention participants showed a significant reduction in depressive symptoms as measured with the CES-D at post-test and 12 mo follow up, but not at 6 months	Post-test:^c^ 0.31 (s)	3
Mackinnon *et al*. (2008) [24]	(BluePages) for community dwelling adults with symptoms of depression	Content: evidence-based medical/psychological depression-information plus weekly telephone calls Type: Psycho-educational website. Duration: 6 weeks. Post-test/follow up points: post-test, 6 and 12 months		Cut-off score: ≥ 16		6 months 0.25 (s) 12 mo:0.37 (m)	2 3
Geisner *et al*. (2006) [22]	To evaluate the efficacy of a brief, mailed personalized feedback intervention designed to alleviate depressed mood	RCT; brief mailed personalized valid feedback (*n *= 89) versus attention placebo controls (thank-you letter; *n *= 88). Format: individual. Content: empathic statement, feedback on test-results and advice. Type: email Duration: 1 session. Post-test/follow up points: 1 month	College students (18 years and older) from West coast public university, USA	Beck Depression Inventory (BDI) Cut-off score: ≥ 14 DSM-IV-based Depression Scale (DDS) Cut-off score not reported	Compared to controls, intervention participants showed a significant reduction in depressive symptoms as measured with the DDS but not with the BDI	BDI: 0.07 (s) DDS: 0.07 (s)	
Jacob *et al*. (2002) [23]	To determine the effect of patient education on outcome of depression	RCT; education intervention (*n *= 34) versus no intervention (*n *= 32). Format: individual. Content: evidence-based medical/psychological depression/anxiety information and advice. Type: leaflets. Duration: 1 session. Post-test/follow up: 2 months	Asian women (18 year and older) in primary care in the UK	General Health Questionnaire (GHQ) Cut-off score: ≥ 3	Compared to controls, intervention participants showed a significant higher recovery rate of common mental disorders as measured with the GHQ (odds ratio: 2.99, 95% confidence interval: 1.03-1.7)	GHQ: 0.61 (m)^d^	4
Kawakami *et al*. (1999) [25]	To examine the effects of mailed advice on reducing psychological distress	RCT; mailed personalized valid feedback and advice (*n *= 81) versus no intervention (*n *= 77). Format: individual. Content: personalized feedback of test-results and advice to reduce psychological distress. Type: email Duration: 1 session. Post-test/follow up: 12 mo	Workers employed in a manufacturing plant in Japan	GHQ Cut-off score not reported	There was no significant difference between controls and intervention participants in GHQ-scores	0.04 (s)	2

^a ^Calculations are between group effect sizes. Where multiple effect sizes for one time point were possible, the largest effect size is reported.

^b ^JQR = Jadad Quality Rating

^c ^s = small; m = moderate

^d ^effect size is based on ITT data

### Study quality

Based on Jadad *et al*.'s [15] criteria, study quality was assessed against three key criteria: randomization; double-blinding; and withdrawals and dropouts. Quality ratings range from 0 to 5, although intervention trials for mental health disorders rarely are rated above 3 as double-blind conditions often cannot be achieved.

### Outcome measures

Primary outcome measures included reduction of depression, anxiety and psychological distress scores as measured on depression, anxiety or psychological distress scales. A second aim was to identify factors which may have contributed to the success of the intervention, such as the setting, the method of delivering psychoeducation, and whether the psychoeducation was based on evidence-based guidelines or research materials.

### Meta-analysis

For each comparison between a psychological treatment and a control group, we calculated the effect size indicating the difference between the two groups at post-test (Cohen's d or standardized mean difference). Effect sizes were calculated by subtracting (post-test) the average score of the psychological treatment group from the average score of the comparison group, and dividing the result by the pooled standard deviations of the two groups. Effect sizes of 0.8 can be assumed to be large, while effect sizes of 0.5 are moderate and effect sizes of 0.2 are small [16].

In the calculations of effect sizes we only used those instruments that explicitly measured symptoms of depression, anxiety or psychological distress. If more than one depression measure was used, the mean of the effect sizes was calculated, so that each study only provided one effect size. If means and standard deviations were not reported, we used the procedures of the Comprehensive Meta-Analysis software (CMA [see below]) to calculate the effect size using dichotomous outcomes. Effect sizes were calculated using the differences between the psychoeducation and the control group immediately at post-test. Follow-up effect sizes could not be calculated because of the small number of data points available. In addition, the follow-up period differed considerably among these studies. Effect sizes were calculated for both completer and intention to treat data, if provided.

To calculate pooled mean effect sizes, we used the computer program CMA (version 2.2.021). As we expected considerable heterogeneity among the studies, mean effect sizes were calculated using a random effects model. In the random effects model it is assumed that the included studies are drawn from 'populations' of studies that differ from each other systematically (heterogeneity). In this model, the effect sizes resulting from included studies not only differ because of the random error within studies (as in the fixed effects model) but also because of true variation in effect size from one study to the next. For continuous variables, we used meta-regression analyses to test whether there was a significant relationship between the continuous variable and the effect size, as indicated with a *Z*-value and an associated *P*-value. As the analysed studies used different measures (both continuous and dichotomous) to indicate effectiveness, one OR was converted into Cohen's *d *effect sizes. The conversion from OR to Cohen's *d *was conducted with the method proposed by Hasselblad and Hedges [17]. It is based on the following formula: Cohen's *d *= v3 * LogOR/π.

The *Q *statistic was calculated as an indicator of homogeneity. A significant *Q *rejects the null hypothesis of homogeneity and shows that the variability among effect sizes is greater than what would likely have resulted from sampling error alone in the primary studies. Additionally, the *I*^2 ^statistic, an indicator of heterogeneity, was calculated; 0% indicates no observed heterogeneity and larger values show increasing heterogeneity, with 25% regarded as low, 50% as moderate and 75% as high [18]. As the standardized mean difference is not easy to interpret from a clinical point of view, we transformed the standardized mean differences into the numbers needed to be treated (NNT), using the formulae provided by Kraemer and Kupfer [19]. The NNT indicates the number of patients that would need to be treated in order to generate an additional positive outcome in one of them [20]. Publication bias was tested by inspecting the funnel plot on primary outcome measures and by Duval and Tweedie's trim and fill procedure [21] which yields an estimate of the effect size after the publication bias has been taken into account (as implemented in Comprehensive Meta-analysis, version 2.2.021).

## Results

### Search results

**Figure 1:**
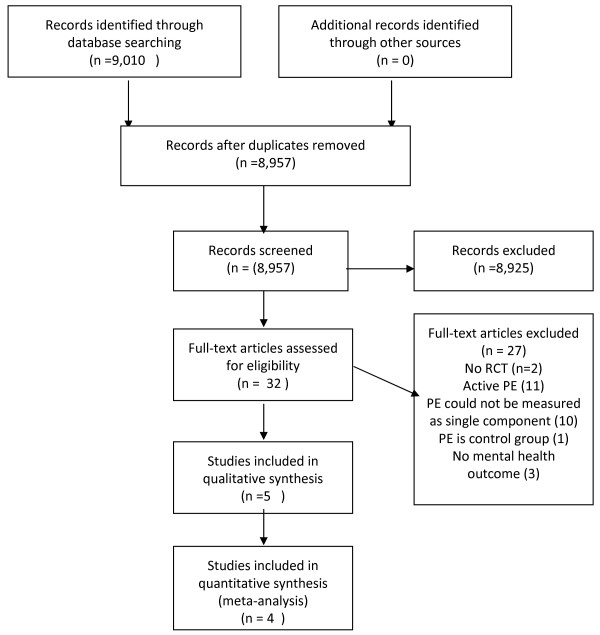
**Flow diagram for passive psychoeducation for depression, anxiety and psychological distress**.

A total of 9,010 abstracts were retrieved, including 436 systematic reviews or meta-analyses. Of these, 32 papers were potentially eligible for inclusion and the full text for each of these papers was retrieved for further screening. Five papers describing four studies targeting depression and psychological distress met the review inclusion criteria [10,22-25]. No randomized controlled trials for anxiety were found. Nine systematic reviews or meta-analyses were further screened for possibly relevant references. On the basis of this screening, 15 full text papers were retrieved for further assessment. However, none were included for final analysis. A flow diagram is shown in Figure 1.

### Characteristics of included studies

Of the five relevant papers, four papers describing three studies [10,22-24] used depressive symptoms or disorders as primary outcome measure, while one study [25] reported psychological distress as an outcome measure (see Table 1). Two studies [10,23] used evidence-based medical/psychological depression/anxiety information; one of them also gave advice [23]. Two studies [22,25] used mailed feedback based on test results and provided advice and one study [23] used leaflets as intervention type. Two papers reporting one study [10,24] used a website. Two studies [10,22] compared the intervention with an attention placebo-control, while two studies [23,25] compared the intervention to no intervention condition. One study [10] recruited participants from the community, one study [23] used primary care participants, one study [25] recruited employees and one study [22] included college students. A total of 694 participants were recruited across all the studies. All included studies used individual rather than group formats. Interventions across all studies ranged from one single email or leaflet to six sessions of psychoeducation. See Table 1 for an overview of the included studies.

### Methodological quality of included studies

The quality of most studies was adequate. Assessors of outcomes and participants were blinded for treatment assignment in only one study. Drop-out rates varied between 4% and 17%. Two studies reported outcomes based on completer analysis [22,25], two describing one trial reported intention to treat data (ITT) data in addition to completer data [10,24] and one study [23] reported an odds ratio based on ITT data. Consequently, except for the converted effect size of Jacobs' [23], all effect sizes are based upon the completer's data.

### Effects of the psychoeducational interventions at post-test and follow-up

#### Depression

All three trials which involved participants with depression found significant reductions (*P *< 0.05) in depressive symptoms or mental health symptoms for the psychoeducation intervention relative to the control on at least one measurement scale and at least one measurement time [10,22,23]. Effect sizes from all outcome variables for these papers ranged from 0.07 (not significant [22]) to 0.61 (significant [23]).

#### Psychological distress

One study which targeted psychological distress [25] found no significant difference (*P > 0*.05) between controls and intervention participants on the General Health Questionnaire ([26])-scores (*d *= 0.04).

**Figure 2:**
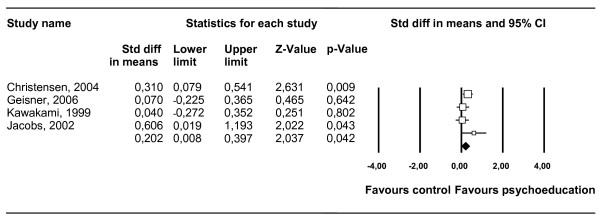
**Standardized effect sizes of passive psychoeducation for depression and psychological distress at post-test**.

We were able to compare a passive psychoeducation intervention with a control group (no intervention, attention-placebo or waitlist) in four comparisons. The pooled standardized-effect size (four studies, four comparisons) for reduced symptoms of depression and psychological distress at post-intervention was *d *= 0.20 (95% confidence interval: 0.01-0.40; *Z *= 2.04; *P *= 0.04, which corresponds with an NNT of 9. Heterogeneity among the studies was not significant (*I*^2 ^= 32.77, Q:4.46; *P *= 0.22). The results of these analyses are summarized in Table 2. The results of depression studies alone are also provided in Table 2. The effect sizes and 95% confidence intervals of the individual contrast groups are shown in Figure 2.

**Table 2:** Meta-analyses of studies comparing the effects of passive psychoeducation for depression and psychological distress at post-test.

Study	*d*	95% CI	*Z*	*I*^2^	Q	P*	NNT
Passive psychoeducation for depression and psychological distress	0.20	0.01~0.40	2.04	32.77	4.46	0.22	9
Passive psychoeducation for depression only	0.26	0.03~0.50	2.17	35.51	3.10	0.21	7

* The *P*-values in this column indicate whether the Q-statistic is significant

CI, confidence interval; NNT, numbers needed to be treated.

Neither the funnel plot nor Duval and Tweedie's trim and fill procedure pointed at a significant publication bias. The effect size indicating the difference in the reduction of depressive symptomatology between the two conditions did not change after an adjustment for possible publication bias (the observed and adjusted effect sizes were exactly the same and the number of imputed studies was zero).

## Discussion

### Main findings

#### Effectiveness of psychoeducational interventions

Results from this meta-analysis of passive psychoeducational interventions for depressive, anxiety and psychological distress symptoms show a small, but significant, effect (*d *= 0.20) on depression and psychological distress in the intervention groups compared to controls.

Similar results on the effectiveness of psychoeducational interventions have been reported in a meta-analysis of single-session personalized-feedback interventions for problem drinking (pooled standardized-effect size was *d *= 0.22; [14]). Active psychoeducation includes materials such as books which describe and teach CBT. Cuijpers *et al*. [12] found that active psychoeducational interventions improved functioning over control conditions. Effect sizes ranged from 0.10 (bibliotherapy compared to individual and group therapy) to 0.82 (bibliotherapy compared to wait list). The effect sizes reported in Cuijpers *et al*.'s review [12] were larger than those found in the present meta-analysis of passive interventions. This is not unexpected, given that the active psychoeducational interventions were all based on CBT techniques, all were guided by a therapist and the duration of the intervention ranged from 4 to 11 weeks. In contrast, the passive psychoeducational interventions included in this review were non-guided, with durations between a single email or leaflet to six sessions of psychoeducation.

Of the four studies investigated, one study which targeted psychological distress [25] did not find significant effects. The reason for this is unclear. One explanation for this finding is that this study used a 12-month post-test in combination with a brief email; it is possible that brief passive psychoeducation is effective directly after the intervention but not sustainable after 12 months. Another study [22] found a significant reduction in depressive symptoms for the intervention group compared to the control group. However, in our meta-analysis, we found a non-significant effect size of 0.07. This apparent discrepancy in findings could be caused by using different statistical analysis to calculate effect sizes, with the effect size reported by the original researchers being associated with an interaction effect and the effect size computed in the current meta-analysis based on a between group effect at post-test. However, since the pooled effect size of this meta-analyses showed a significant effect size of *d *= 0.20, we can conclude that passive psychoeducational interventions can be effective in reducing depressive symptoms.

Passive psychoeducational interventions (for example, leaflets) can be readily disseminated through general practice and therefore are capable of reaching a large number of people at relatively low cost. Psychoeducation has the potential to target the public and perhaps influence individuals at risk of suicidal behaviour [27]. To date, little literature exists on the effect of psychoeducation for modifying suicidal behaviour and, to our knowledge, no specific studies have been conducted using passive education alone. Existing reviews of suicide prevention programmes which incorporate a psychoeducational component provide ambiguous results, both beneficial [28] and harmful [29]. Passive psychoeducation has the potential to influence suicide rates [27] by improving treatment adherence [30], by increasing knowledge and or by improving attitudes to mental illness and suicide, but this requires systematic research.

Although the effect sizes in this meta-analysis are not large, some current reviews of antidepressant medication for moderately depressed patients (for example [31]) also yield small effect sizes. This places in context the potential role of psychoeducation as a legitimate, inexpensive intervention, with minimal or no side effects.

#### Factors influencing effectiveness of psychoeducational interventions

Given the small number of studies identified, it is difficult to isolate any factors that might influence the effectiveness of psychoeducation. However, we did note that somewhat larger between-group effect sizes were found in the psychoeducational interventions using evidence-based medical/psychological depression/anxiety information (0.25 to 0.61) compared to feedback on test-results and advice (0.04-0.07), suggesting that content of intervention might influence effect size. Other factors such as type of delivery (website, leaflets or email) were not found to be strongly linked to outcome, a finding that suggests that the specific content and written delivery mode (website, leaflets or email) may not be critical. However, because of the paucity of the included studies this observation would need to be tested further.

#### Sustainability of results

Two studies reported follow up data no longer than 2 months after the intervention. Sustainability of these results after longer periods is unclear. One study found benefits were retained over a period of at least 12 months [24] and one study did not [25]. It might be that such factors as content of the information, type of delivery or length of the intervention influence outcome, but as mentioned earlier, this would need to be tested further.

### Limitations

The limitations of this review need to be acknowledged. First, only a small number of studies were eligible for inclusion in this review. Due to variability in population, method of recruitment, inclusion criteria, content and type of intervention, it was difficult to make comparison between studies. Therefore, factors influencing the effectiveness of an intervention were difficult to determine. Second, one study reported that a number of the participants who were included in the psychoeducation group were concurrently taking psychotropic medication. However, since some of the participants who were included in the control condition were also concurrently taking medication, it is unlikely that pharmacotherapy explains the pattern of results. Third, three reported effect sizes in this meta-analysis were based on the completer's data and one effect size was based on ITT data. Completer data is likely to yield higher effect sizes as those retained in the study may be more likely than those who dropout to show positive effects. However, this was not the case in the present study with the highest effect size associated with an ITT design. Two papers [10,24] describing one trial provided effect sizes based on completers data as well as ITT data. This effect size (0.29) was not very different from the effect size (0.31) based on completers data. Thus, although it is not optimal to combine studies with different designs and biases, we think it unlikely that combining design types biased findings in this instance. Finally, we only included studies from peer-reviewed, English language journals. However, the effect of language bias minimally impacts the conclusions of systematic reviews [32].

## Conclusions

This meta-analysis indicates that brief passive psychoeducational interventions targeting depression and/or psychological distress symptoms can be effective, albeit that effect sizes are typically small. Passive psychoeducational interventions are relatively easy to implement and can be applied by non professionals. These interventions may be viewed by consumers as less stigmatizing when delivered through a website, email or leaflet. Because they can be applied immediately, and are unlikely to be expensive, they may offer a first step intervention for those experiencing anxiety or depression. Psychoeducation could be readily incorporated into primary care, general practice and stepped care models.

Psychoeducational interventions are often used as the content for attention-placebo control arms in randomised controlled trials [33,34]. As such, they may reduce the likelihood of detecting a true effect in the intervention arms of the trial. Therefore, alternatives to psychoeducational intervention as control groups (for example, attention placebo) are recommended, in order to avoid bias in study outcomes. Finally, more research is needed to further examine the factors (for example, method of delivering, length of intervention) influencing the effectiveness of psychoeducational interventions. Furthermore, there is a need for randomized controlled studies focusing on the effectiveness of passive psychoeducational interventions for reducing anxiety symptoms.

## Abbreviations

CBT: cognitive behavioural therapy; CMA: comprehensive meta-analysis; IPT: interpersonal therapy; ITT: intention to treat; NNT: numbers needed to be treated.

## Competing interests

The authors declare that they have no competing interests.

## Authors' contributions

TD, KG and HC contributed to the design of the study. TD carried out the screening procedure and drafted the manuscript. TD and PC performed the statistical analysis. TD, KG and HC contributed to the further writing of the manuscript. All authors read and approved the final manuscript

## Pre-publication history

The pre-publication history for this paper can be accessed here:

http://www.biomedcentral.com/1741-7015/7/79/prepub
